# Stigma, Culture, and Care: A Quantitative Analysis of Mental Health Treatment-Seeking Behavior Among Hondurans

**DOI:** 10.7759/cureus.97857

**Published:** 2025-11-26

**Authors:** Chris Sancilio, Abby R Tavallai, Lauren J Burch, Skyler Sorkin, Elie Bachour, Maison D'Amelio, Greg Jacobs

**Affiliations:** 1 Research, Alabama College of Osteopathic Medicine, Dothan, USA; 2 Clinical Sciences, Alabama College of Osteopathic Medicine, Dothan, USA

**Keywords:** anxiety, depression, epidemiology, international health, mental health, rural medicine

## Abstract

Objective: During a medical service trip to rural Honduras, novel data were recorded, which indicated that Honduran citizens have a negative outlook on seeking treatment for mental health disorders. These data are one of a kind with very little research being done on mental health within third-world countries like Honduras, and subsequently a notable gap in the literature on the receptivity of citizens towards treating mental health conditions. The objective of this study is to use previously collected data on depression and anxiety in Honduras and quantify the attitudes of citizens, specifically towards treatment, with a concurrent analysis on the confounding variables surrounding mental health in Honduras as a whole.

Methods: Data were collected in the Santa Barbára region of Honduras spanning over 2000 patients seen by the team involved in the medical aid. Results were narrowed down to 85 total patients who fulfilled the inclusion criteria. The survey consisted of four questions that followed up after the completion of a complete medical history, with a focus on openness and accessibility towards treatment of mental health disorders with associated variables. Data was analyzed using Pearson’s correlation coefficient, two-tailed T-Test assuming equal variances. This study was designed in alignment with the osteopathic philosophy that health is influenced by the interrelationship of mind, body, and spirit and the importance of treating the whole person. In line with this approach, the survey methodology incorporated cultural, psychosocial, and environmental variables that may influence mental health treatment-seeking behavior. Internet use, social support, household structure, and perceived stigma were measured as reflections of determinants of health beyond clinical treatment.

Results: The results of the initial survey asking if patients would seek professional help for a mental health disorder showed a 52.9% majority in favor of denying treatment from a professional. Conversely, 94.1% of survey respondents chose they were open to talking to friends about mental health disorders. Using a Pearson correlation coefficient, the results showed an increase in the standard deviation of treatment-seeking behavior of 0.32 per hour of internet usage time showing a positive correlation between the two variables above the 0.30 threshold standard deviations for strong correlation. Using a two-tailed t-test, it was found that the results of the Pearson Correlation Coefficient were statistically significant with P = 0.000028. Additional survey results showed that 42.1% of respondents would not select treatment based on cost, also the same percentage did not specify a reason as to why they would not seek out treatment. 5.3% of respondents would not seek out treatment due to work conflicts. Lastly, 10.5% of respondents would not seek out treatment due to distance.

Conclusion: Through this study, we were able to identify the attitudes of Honduras towards seeking out treatment when faced with mental health conditions. Novel data introduced in this study show a statistically significant positive correlation between social media usage, and mental health treatment-seeking behavior. With the data showing a majority of Hondurans declining the option of seeking out professional treatment for mental health disorders, if they were faced with one, we strongly encourage further research into expanding availability of care for mental health disorders in Honduras.

## Introduction

Mental health is a fairly new branch of medicine when compared to physical medicine, with the first ever mention of depression in the modern sense of the condition cited back to 1947 in the National Library of Medicine, where Macalman first described the condition as “endogenous depression” [1]. To our knowledge, this is the first incidence of depression being labeled as pathological. Since that time, with an exponential increase around the 1990s, depression and anxiety have become a well-researched concept. We found, however, that the literature on depression in developing countries is far and few between, with only 64 mentions of depression in Honduras in the same database. Most of these data were recorded following the COVID-19 pandemic, when mental health took a spotlight within the global medical scene. Mental health in the rural regions of Honduras has never been exclusively documented, and through the works of a United States-based organization called Action for Education [2], we have access to novel data that gives insight into what the populations of that demographic look like. Wulsin et al. gave us a first-ever sample of depression in Honduras as a whole by evaluating the feasibility of using a PHQ-9 screen for depression in populations that had never been exposed to research before [3]. This pilot study found an estimated prevalence of 15% of citizens in Honduras to be positive for depression. After reviewing these data against the new database from Action for Education, collected from 2023-2025, we hypothesized the levels to not only be much higher than that, but also the attitudes towards mental health to be aversive. Following the initial study in 2002, Wulsin et al. conducted a follow-up study in 2010, where the level had increased up to 17.6% among mothers and children in Honduras [4]. Since then, minimal data have been collected on the national level regarding rural demographics and, more specifically, down to variables associated with treatment-seeking behavior. Technology has often been cited in the literature as being associated with mental health conditions. Andreasson et al. found a positive correlation between the usage of technology, such as video games and social media, and mental health conditions, including depression and anxiety [5]. On the opposite end of the spectrum, however, studies have been done measuring the usage of technology with a positive correlation towards telemedicine visits [6]. These data presented two sides to a story, but bridging the gap between mental health and treatment-seeking behavior, with regard to technology, has yet to be quantified in Honduras.

Access to medical care in Honduras is on par with other developing countries in Central America, with levels far below what is available in the United States. Studies have been done on the accessibility of care for chronic medical conditions in Honduras, such as diabetes, where it has been shown that there is a severe lack of availability for even basic monitoring of conditions [7]. Another shocking study found that there is an extreme lack of care available for emergent healthcare (EMS) as well as chronic care. Individuals are urged by the government to actually avoid using the public systems in place for EMS due to the long wait times and lack of a central dispatch center, even going as far as stating they might not ever receive the desired care [8]. With regard to mental health, the statistics for medical professionals specializing in mental health are very slim, with Brito et al. finding that there is one psychologist per one hundred thousand citizens in the country [9]. 

Systemic barriers to treatment for depression and anxiety in Honduras are not often thought about in the United States, with mental health taking a backseat to physical medicine, as evidenced by the lack of research involving the subject. The primary objective of this study is to quantify attitudes towards seeking professional mental health treatment among adults living in rural Santa Barbara, Honduras, as well as to assess how internet use, measured in hours per week, relates to willingness to seek care for depression and anxiety. In addition, perceived barriers to treatment, such as cost, lack of transportation, and limited accessibility, were explored. We hypothesize that increased internet use is associated with a greater willingness to pursue professional mental health treatment, which reflects a shift in not only awareness but also attitudes within these communities. Using a cross-sectional survey and correlational analysis of data collected between 2023 and 2025, this study aims to contribute a more accurate depiction of how mental health disorders are perceived in rural Honduras. These findings underline the importance of continued research into this topic with the hope of overcoming the barriers to availability of mental health care faced by Hondurans of rural regions, and the country as a whole.

## Materials and methods

Data collection

The retrospective data being used in this study were collected from an independent database via RedCap Electronic Medical Records, exclusively on service trips to rural regions of Honduras during trips spanning 2023-2025 by an organization called Action for Education, based out of the United States. The EMR contains over 2000 patients of all age groups and demographics who were seen by the primary care physicians during the medical trips. Research was collected by the appropriate staff within the organization using inclusion and exclusion criteria. This study pulls data from 85 patients of various demographics within close proximity, all from the Santa Barbára region of Honduras, who chose to fill out the voluntary survey regarding their technology usage, attitudes towards seeking treatment from friends, family, and medical professionals, as well as barriers towards mental health care. Participation in the study was voluntary and did not impact the remainder of their medical care, and verbal informed consent was obtained from all participants prior to the completion of the survey. Approval for this study was obtained through the Honduran hospitals, Hospital Leonardo Martinez Valanzuela and the Juan Manuel Galves Public Hospital, along with the mayor and local Honduran Ministry of Health in Santa Barbara, Honduras.

Demographics

Inclusion criteria for the study participants included patients who were willing and agreeable to participating in research with proper consent. Exclusion criteria were patients under the age of 18, as well as patients who were not able to communicate properly with the local translators to complete at least 50% of the comprehensive survey collected separately from the study’s survey.

Instrumentation

The collection of the data was done by appropriate staff trained in collecting the data in a professional manner, using a team of Honduran translators to assist. The data itself was collected directly onto iPads into the EMR anonymously using only numerical identifiers to preserve the identity of the individuals involved. No other identifying data was utilized for the purpose of this study besides that which is included in the methods.

Survey design

The voluntary survey administered to the participating patients included a series of four questions previously inputted into the Project RedCap Electronic Medical Record under the Records page, by appropriately trained physicians from the United States. The patient research was conducted individually in private, while waiting for their prescription to be filled, following a visit with one of the physicians, or when they were waiting to be seen by the physicians. Surveys were conducted using a translator, with the question being spoken in English by the administrator of the survey to the translator, who then spoke the question in Spanish to the patient, who returned their answer in Spanish as well, which was then repeated back to the administrator in English. The questions were asked sequentially with no alternative screening or testing done in between each question. The survey questions are as follows, first in English, then in Spanish.

Question One

How many hours per week do you spend on the internet on average?

¿En promedio, cuántas horas por semana pasas en el Internet?

Question Two

Do you feel comfortable talking to any close family members, friends, or acquaintances about feeling overwhelmed, down, or hopeless?

¿Sientes cómodo hablando con familia, amigos, o conocidos de sentirse abrumado, deprimido, o desesperado?

Question Three

Would you seek treatment (therapy or medication) for strong emotions, like depression/excessive sadness or anxiety, if you were given the resources and opportunity?

¿Buscaría tratamiento (terapia o medicación) para emociones fuertes, como depresión/tristeza excesiva o ansiedad, si tuviera los recursos y la oportunidad?

Question Four

What would prevent you from seeking help from a doctor or therapist for a mental health problem? 

¿Qué le impediría buscar ayuda de un médico o terapeuta para un problema de salud mental?

Data analysis

The data were pulled from the EMR manually and compiled into an Excel version 16.96.1 spreadsheet using the numerical identifiers of each patient. The total number of patients who filled out some, or all, of the voluntary survey was 85. Some of the patients, however; did not answer the entirety of the survey for variables discussed within the results and discussion session. Thematic analysis was conducted to examine and consolidate the responses to the open-ended questions, using an inductive approach to identify similar answers and refine them into broader groups for coding purposes. All authors agreed upon the consolidation of the responses to match into the thematic group appropriately, in order to more accurately depict the results of the findings. Some of the responses were consolidated into broader categories to fit into the binary coding of the data, such as a response for question four being “Too expensive” consolidated into the section “Cost”. The data was analyzed for significance using a two-tailed t-test assuming equal variance, the Pearson Correlation Coefficient for standard deviation of correlation between the findings, as well as and elucidation of the findings through three separate visuals, including two conjoined bar graphs to visualize the quantification of yes/no responses, a graph to show prevalence of the differing qualitative responses for perceived barriers to care, and a distribution of internet usage per week.

## Results

Response rate

**Figure 1:**
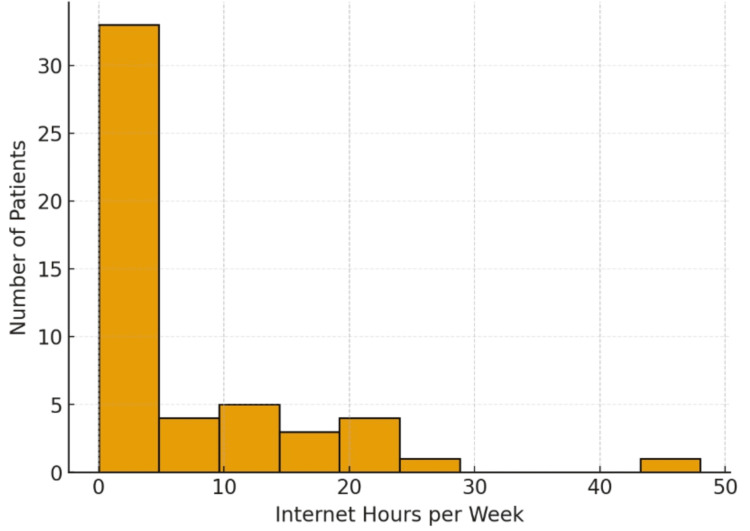
Graph showing the distribution of reported internet usage per week in hours. Internet Usage per Week

**Figure 2:**
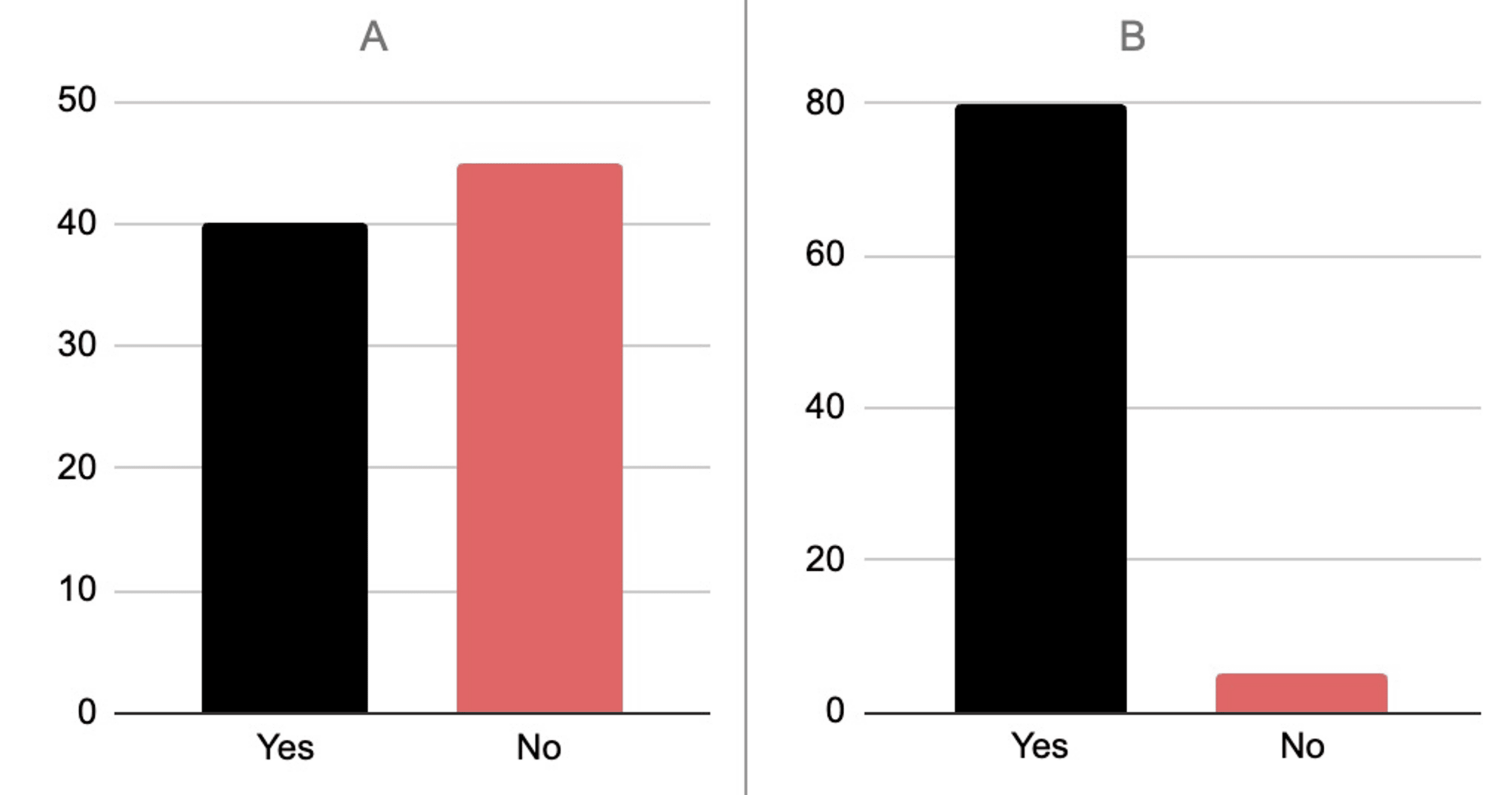
Bar graphs reporting the responses to whether or not patients would seek out treatment from a medical professional for a mental health disorder (A), as well as if the patient would feel comfortable talking to family if faced with a mental health disorder(B). A. Willing to Seek Professional Treatment B. Comfort in Talking to Family about Mental Health Treatment

**Figure 3:**
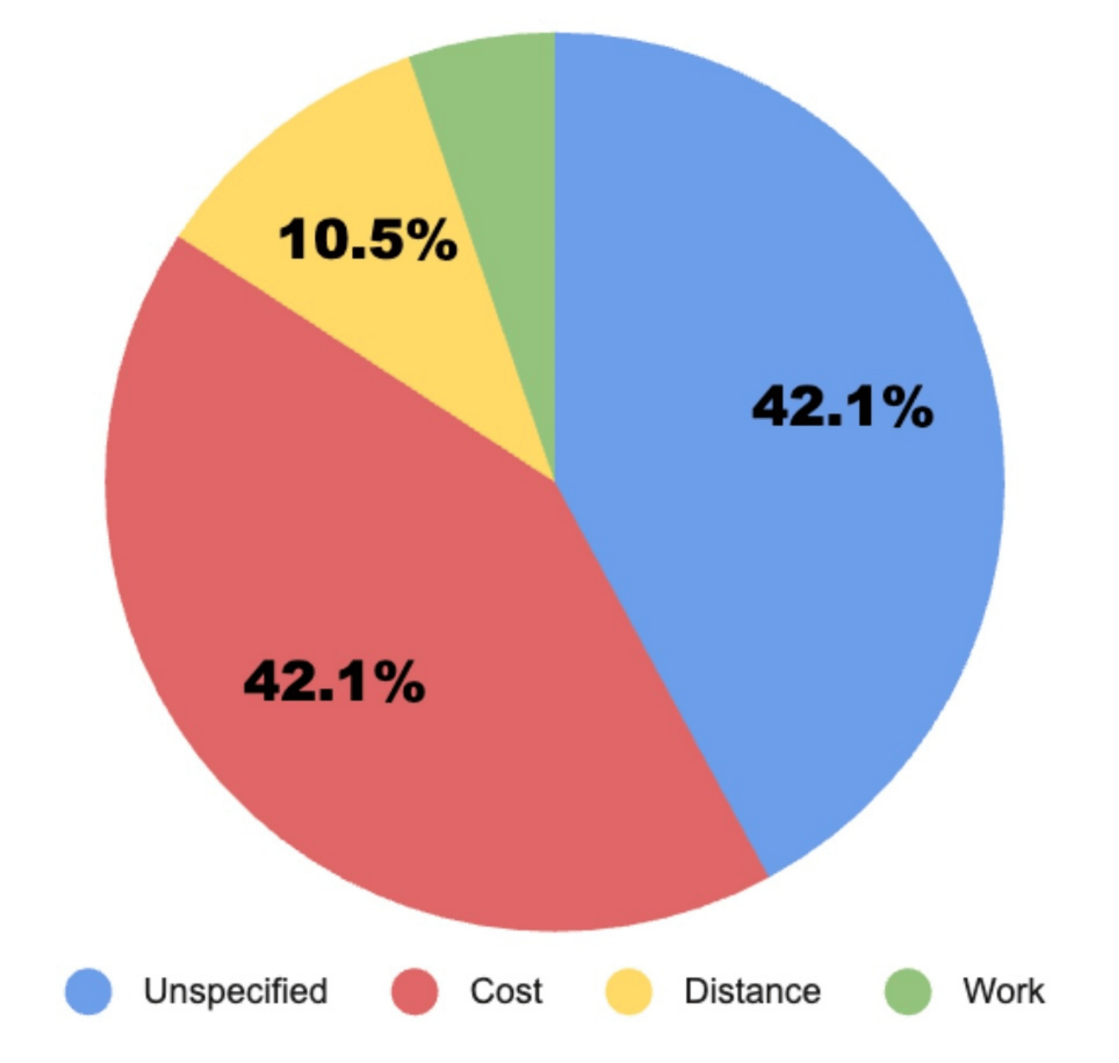
Chart showing 42.1% of respondents would not pursue treatment based on both cost, and also the same percentage had no reason as to why they would not seek out treatment. 5.3% of respondents would not seek out treatment due to work conflicts. Lastly, 10.5% of respondents would not seek out treatment on the account of distance. Reported Barriers to Seeking Treatment

The results of the survey questions were recorded individually with a variance in participation for each response. Question one, regarding internet hours used each week, had a 38.8% response rate with 33 patients responding to the given question (Figure 1). Question two, regarding openness to talking with friends and family about mental health, had a 100% response rate with all 85 patients answering the question. Question three, regarding openness in seeking treatment from a professional for a mental health problem, also had a 100% response rate with all 85 patients responding to the given prompt (Figure 2). Question four, only asked to those who answered no to question three, in regards to the reason they would not seek treatment, achieved a 42.2% response rate out of the 45 patients who answered no to question three (Figure 3). 

Internet usage per week

The independent quantitative variable within the survey was hours per week spent on the internet (phone or computer) by the Hondurans. This question was used for translation into interconnectedness with others and a foundation for how socially we consider the patient to be outside of face-to-face interactions. We did not take into account other modalities of social interconnectivity, such as mailing, faxing, etc. The results of the questioning regarding internet usage, compared to the same patients’ answers to questions regarding seeking treatment, showed that there is a strong positive correlation between internet hours per week and willingness to seek treatment. For every standard deviation increase in hours per week, there is a 0.32 standard deviation increase in willingness to seek treatment. Using a two-tailed t-test, it was found that the results of the Pearson correlation coefficient were statistically significant with p = 0.00002787795772.

## Discussion

Mental health in developing countries is often a topic that falls below the radar, with priorities being in other areas of healthcare. When it comes to mental health, notably depression, there is a large focus on topics such as perinatal depression, or depression among young mothers in developing countries, along with the inability for timely screening and diagnosis [10]. This focus within the topic of mental health is very important, but it also identifies a large gap in the literature concerning the mental health of other demographics within the same population. Among the studies previously conducted, such as two studies by Reuland et al. and Golding et al. in 2009 and 1991, respectively, it was found that Hispanic cultures in Central America often have a different outlook and definition of depression altogether, with a coinciding lower willingness to disclose these topics as well [11,12]. Identifying mental health conditions such as depression and anxiety presents a new frontier for healthcare in rural developing countries, and the objective of this study is to help identify trends within these communities associated with treatment-seeking behavior.

Limitations to this manner of study were illustrated by Reuland et al. in the 2009 study, which stated that the translation of common mental health disorders, such as depression and anxiety, did not have the same implications that it does in the Western world [10]. We know from previously conducted studies in low-resource countries that with the use of standardized screening tools such as the PHQ-9 and GAD-7, it still remains possible to identify patients who exhibit the level of symptoms of depressive and anxiety disorders [13]. With a reliable measure, despite language barriers, we are able to examine further behaviors that could help to assist in the treatment and prevention of mental health disorders. 

Social norms and expectations are a critical part of Honduran society, but also present a barrier by which Honduran’s feel pressured to maintain roles. A 2023 study in Honduras presented qualitative data to identify barriers that women face when facing interfamilial issues. This study showed that a significant portion of these women felt that their societally imposed position within the family dictated their willingness to seek treatment [14]. This study helps us to establish that family and community play a large role in the willingness of Hondurans to seek treatment for conditions that might not be accepted as normal by the larger community. Aboaja et al. endorse the idea that Latin-American populations were more likely to discuss mental health in a personal setting rather than pursue formal care [15]. This becomes evident in the novel data introduced in this study, which showed a significant decrease in the percentage of Hondurans who were willing to seek treatment for mental health conditions from a professional, when compared to those who were willing to seek help from friends and family. A limitation to these results, however, is the sample size. With only 85 participants, the generalizability of the findings is limited.

With the ever-increasing usage of technology in all countries around the world, a critical examination must be made to determine which areas of society will be helped by technology or hurt by it. In a landmark study by Piette et al. in 2010, a claim was made that with an increasing use of technology, a patient is more likely to seek and accept medical care via telemedicine [6]. This study builds an important foundation for studies such as this one that seek to answer questions within domains of medicine that might be helped by technology. The data presented within this study highlight novel findings of a positive correlation between treatment-seeking behavior and the usage of technology on a daily basis. Despite a positive correlation, we cannot conclude that increased usage of technology is causative of increased treatment-seeking behaviors for mental health disorders; rather, it remains an association that highlights the need to expand the scope of future research.

Among the forefront of problems faced within low-resource populations is the lack of medical care of all types. There is a significant lack of availability of mental healthcare within developing countries, with the cited number being one psychologist per 100,000 people in Honduras [9]. This number compares to the United States, where it is estimated to be 182 mental health professionals per 100,000 citizens [16]. This marked discrepancy of mental health professional care reinforces a theme that emerges in many different sectors of healthcare, but with a clear implication of a lack of access to treatment when facing mental health disorders in Honduras and other developing countries.

## Conclusions

After an analysis of the current literature regarding the current conditions for mental health care in Honduras, it was evident that the landscape for seeking treatment for depression, anxiety and other disorders is significantly affected by variables such as stigma and culture. With a large emphasis placed on societal norms, mental health was shown to take a secondary role in the landscape of healthcare within these regions. Of the data collected from the patients in Santa Bάrbara, Honduras we found that there is a statistically significant correlation between internet usage per week and willingness to seek treatment for mental health disorders such as depression and anxiety, although important to note this correlation may not indicate causation. This positive correlation points towards an attitude that helps to break the longstanding stigma associated with mental health in countries such as Honduras, and surrounding regions, where disorders such as depression and anxiety generally do not even contain the same weight that they do in the United States. The overall data still shows a majority of the respondents would still decline to seek out professional treatment when faced with one of these conditions. We strongly encourage more research on the stigma behind mental health, and what can be done to change it, in countries such as Honduras. We also seek to call more awareness to the issues presented in this study and ultimately work towards increasing the availability of care to those who are choosing to seek it out in Honduras.
